# Reducing time to differentiated service delivery for newly-diagnosed people living with HIV in Kigali, Rwanda: a pilot, unblinded, randomized controlled trial

**DOI:** 10.1186/s12913-024-10950-z

**Published:** 2024-04-30

**Authors:** Jonathan Ross, Kathryn Anastos, Sarah Hill, Eric Remera, Gallican N Rwibasira, Charles Ingabire, Francine Umwiza, Athanase Munyaneza, Benjamin Muhoza, Chenshu Zhang, Denis Nash, Marcel Yotebieng, Gad Murenzi

**Affiliations:** 1 Albert Einstein College of Medicine, 3300 Kossuth Avenue, Bronx, NY 10467 USA; 2 Division of HIV, STIs and Viral Hepatitis, Rwanda Biomedical Center, Kigali, Rwanda; 3 Research for Development (RD Rwanda), Kigali, Rwanda; 4 Institute for Implementation Science in Population Health, City University of New York, New York, 10027 USA

**Keywords:** HIV, Differentiated service delivery, New diagnosis, Sub-Saharan Africa, Rwanda

## Abstract

**Background:**

Differentiated service delivery (DSD) programs for people living with HIV (PWH) limit eligibility to patients established on antiretroviral therapy (ART), yet uncertainty exists regarding the duration on ART necessary for newly-diagnosed PWH to be considered established. We aimed to determine the feasibility, acceptability, and preliminary impact of entry into DSD at six months after ART initiation for newly-diagnosed PWH.

**Methods:**

We conducted a pilot randomized controlled trial in three health facilities in Rwanda. Participants were randomized to: (1) entry into DSD at six months after ART initiation after one suppressed viral load (DSD-1VL); (2) entry into DSD at six months after ART initiation after two consecutive suppressed viral loads (DSD-2VL); (3) treatment as usual (TAU). We examined feasibility by examining the proportion of participants assigned to intervention arms who entered DSD, assessed acceptability through patient surveys and by examining instances when clinical staff overrode the study assignment, and evaluated preliminary effectiveness by comparing study arms with respect to 12-month viral suppression.

**Results:**

Among 90 participants, 31 were randomized to DSD-1VL, 31 to DSD-2VL, and 28 to TAU. Among 62 participants randomized to DSD-1VL or DSD-2VL, 37 (60%) entered DSD at 6 months while 21 (34%) did not enter DSD because they were not virally suppressed. Patient-level acceptability was high for both clinical (mean score: 3.8 out of 5) and non-clinical (mean score: 4.1) elements of care and did not differ significantly across study arms. Viral suppression at 12 months was 81%, 81% and 68% in DSD-1VL, DSD-2VL, and TAU, respectively (*p* = 0.41).

**Conclusions:**

The majority of participants randomized to intervention arms entered DSD and had similar rates of viral suppression compared to TAU. Results suggest that early DSD at six months after ART initiation is feasible for newly-diagnosed PWH, and support current WHO guidelines on DSD.

**Trial registration:**

Clinicaltrials.gov NCT04567693; first registered on September 28, 2020.

**Supplementary Information:**

The online version contains supplementary material available at 10.1186/s12913-024-10950-z.

## Introduction

In recognition of an increasingly diverse set of needs for the millions of people living with HIV (PWH) on antiretroviral therapy (ART), in 2016 the WHO recommended implementation of differentiated service delivery (DSD) models [1]. Such approaches are feasible, acceptable, and achieve equivalent or improved retention in care and viral suppression compared to standard treatment [2–7]. Many countries have adopted facility-based DSD models allowing PWH established on ART to be seen less frequently for clinical assessments (e.g. annually or semiannually instead of quarterly) and receive multi-month ART dispensations (e.g. quarterly or semiannually instead of monthly); some have modified eligibility for DSD in response to the COVID-19 pandemic [8].

DSD programs largely limit eligibility to patients established on ART, yet this status is not well-defined. Prior WHO guidelines (in place at the time of this study) defined established as receiving ART for at least one year with two consecutive suppressed viral loads (VLs); [1] this definition was recently updated to receiving ART for at least six months with at least one suppressed VL [9]. Among facility-based DSD programs in sub-Saharan Africa (SSA), heterogeneity exists with respect to criteria for duration on ART and number of suppressed VLs necessary to be considered established on ART[ 5,10–12]. To date, individuals newly initiating ART have largely been excluded from DSD models [13], and no studies have empirically compared outcomes of newly-diagnosed PWH who transition to facility-based DSD models after shorter intervals in care or fewer VL measurements compared with the current standard of care.

Modifying the definition of established on ART to decrease the time on treatment before beginning DSD could theoretically reduce the number of patient visits and number of VL tests required, thereby decreasing the overall burden faced by patients and health systems. However, implementing DSD earlier in patients’ treatment may not provide them with the necessary support to achieve or maintain viral suppression. Moreover, entering DSD models in the first few months of treatment may not be feasible at a time when patients may not yet be virally suppressed, and may not be acceptable to patients or healthcare providers who may desire more intensive support at this early stage.

We therefore conducted a three-arm, pilot randomized controlled trial (RCT) in Rwanda to explore the impact of two facility-based DSD strategies: (1) reducing the time to entry to DSD from 12 to 6 months after ART initiation and (2) reducing from two to one the number of suppressed VL measurements required to enter DSD. Our objectives were to determine whether these less-intensive DSD models were feasible in the context of current Rwandan HIV guidelines, whether they were acceptable to patients and providers, and whether they would negatively impact viral suppression at 12 months. We hypothesized that the DSD models would be feasible, acceptable and would result in non-inferior rates of viral suppression compared to the standard of care.

## Methods

### Trial design

This was a three-arm, pilot RCT enrolling newly-diagnosed PWH in Kigali, Rwanda (NCT04567693). A full description of the trial was previously published [14]. Briefly, study participants were randomized 1:1:1 to either: (1) entry into DSD at 6 months after ART initiation after one suppressed VL; (2) entry into DSD at 6 months after ART initiation after two consecutive suppressed VLs; (3) treatment as usual (quarterly clinical appointments and monthly ART pick-ups) until the end of the study. Study enrollment began on October 20, 2020 and ended on May 4, 2021 when the target sample size was reached.

### Setting

To optimize service delivery under Treat All, Rwanda introduced facility-based DSD models to align services with the variable needs of different groups of PWH. Stable PWH (adults on ART for ≥ 12 months with two consecutive suppressed VLs) collect ART quarterly (rather than monthly) and attend clinical appointments with a nurse quarterly (adolescents, key populations, and patients co-infected with tuberculosis or hepatitis; “Stable B”) or semiannually (all others, “Stable A”). Individuals in the unstable category (newly-diagnosed PWH on ART for < 12 months, pregnant or lactating women, patients with mental health disorders and PWH who are not virally suppressed) must visit the clinic monthly for ART pick-up and adherence assessment and attend clinical appointments quarterly. Participating health facilities for this study included three public facilities located in Rwanda’s capital, Kigali that together provide primary HIV care to approximately 6000 PWH, including approximately 300 newly-diagnosed patients enrolling in care annually.

### Participants and recruitment

Health facility nurses informed potentially eligible patients about the study during a routine appointment; those interested in participating verbally consented to being contacted by study staff, who screened them for eligibility and offered enrollment. We included individuals who at the time of study enrollment were: (1) ≥ 15 years at enrollment, (2) newly-diagnosed with HIV within 6 months, (3) initiated HIV care at a participating study health facility within 30 days, and (4) initiated ART. Participants were excluded if at the time of study enrollment they: (1) planned on moving away from the Kigali area during the duration of the study, (2) were pregnant or lactating, (3) were coinfected with tuberculosis and had not completed treatment, (4) had a documented severe mental health or substance use disorder, or (5) could not provide informed consent. Given available resources for this pilot study (e.g. research staff time, participant incentives, laboratory investigations), we planned to enroll 90 participants.

### Interventions

**Fig. 1:**
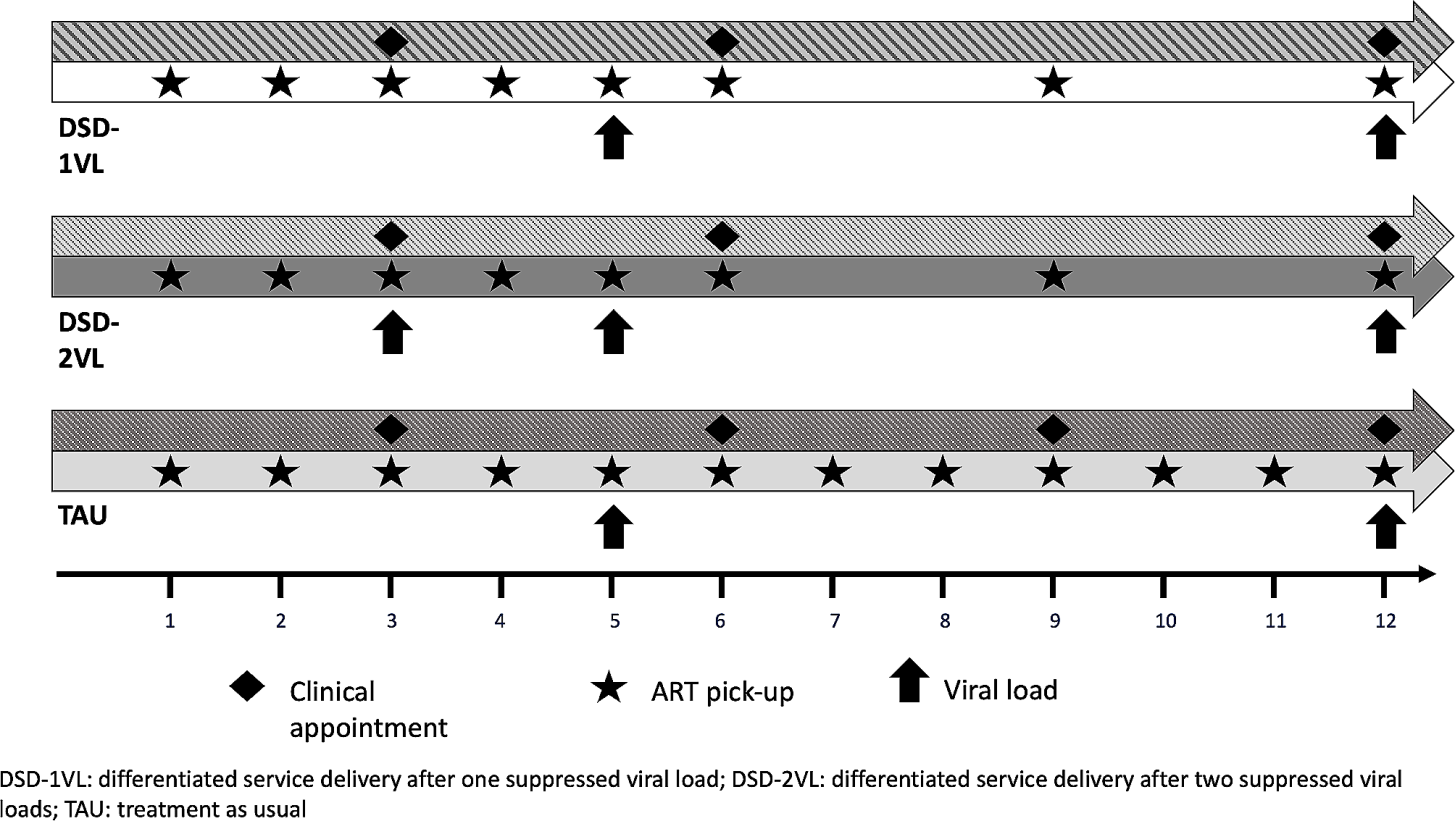
Schedule of clinical appointments, ART pick-ups and viral load monitoring by study arm

For the first six months after ART initiation, all study participants were scheduled for clinical appointments and ART pick-ups based on national HIV guidelines. Participants were randomized within 1 month of ART initiation to one of three study arms (Fig. 1) in a 1:1:1 ratio:

- *Early DSD, one VL (DSD-1VL)*: VL measured at 5 months after ART initiation; if suppressed, participants were eligible to enter the DSD schedule beginning at 6 months after ART initiation.
- *Early DSD, two VLs (DSD-2VL)*: VL measured at 3 and again at 5 months after ART initiation; if both were suppressed, participants were eligible to enter DSD beginning at 6 months after ART initiation.
- *Treatment as usual (TAU)*: VL measured at 5 months after ART initiation; participants remained on the guideline-based appointment schedule of quarterly clinical appointments and monthly ART pick-ups for the duration of the study.

For participants in the intervention arms, the decision to advance to a DSD schedule was ultimately based on clinical assessment by care providers, who were free to override the study assignment.

### Assignment of study arm

After providing informed consent, participants were randomized in blocks of 6 with 1:1:1 allocation across arms. Randomization was computer generated by the study statistician and stratified by age (< 24 vs. ≥ 24 years) and health facility. Research staff used the randomization function in REDCap V.10.0.16, 2020 (Vanderbilt University), to assign study arms. Participants in DSD-1VL and DSD-2VL who became pregnant or developed comorbidities (e.g. tuberculosis) that made them ineligible for DSD crossed over to TAU per the study protocol.

### Outcomes

Feasibility of the two DSD models was measured through examining the proportion of participants assigned to intervention arms who successfully entered DSD, the proportion assigned to intervention arms who crossed over from the assigned study arm to another arm because of change in clinical status, and the mean number of days between encounters among participants in DSD versus non-DSD over months 6–12 of the study.

Acceptability was measured using a 10-item survey of satisfaction with clinical (overall service, explanation from providers, drug availability, medication cost, service cost, staff attitude, privacy) and non-clinical (waiting time, time with provider, cleanliness) elements of healthcare derived from a national impact evaluation in Rwanda [15]; participants rated each element on a Likert scale from 1 (very unsatisfied) to 5 (very satisfied). Additional measures of acceptability included review of instances in which clinicians overrode the experimental assignment and review of adverse event logs.

The primary effectiveness outcome for this study was 12-month viral suppression, measured as the proportion of participants in each arm whose 12-month VL was < 200 copies/ml. A secondary effectiveness outcome, appointment attendance, was defined in the protocol as the proportion of participants in each arm who attended all scheduled clinical appointments and ART pick-ups during the study. We planned to ascertain appointment attendance through review of participants’ medical files at the conclusion of the study. During medical file review, it became apparent that scheduled appointments (i.e. next planned) were inconsistently documented. We therefore were unable to accurately ascertain this outcome.

### Covariates of interest

Additional variables of interest included: ART adherence, measured as the number of days of ART missed in the prior 30 days (self report); quality of life, measured using the EuroQOL-5 Dimension-5 Levels visual analog scale of self-rated health [16], and anticipated, enacted and internalized stigma, measured using a modified version of the HIV Stigma Scale [17] and the HIV/AIDS Stigma Instrument PLWA Scale [18]. 

### Data collection and analysis

Data were collected through participant surveys, laboratory tests and medical record review. Interviews were conducted in Kinyarwanda by staff with responses entered directly into REDCap. Venous blood specimens were collected at study entry, 3 months (for the DSD-2VL arm only), 5 months and 12 months after ART initiation. VL measurements were performed using the Abbott Allinity m instrument, with a lower limit of detection of 20 copies/mL. At the end of the study, participant medical files were reviewed, with clinical appointment and ART pick-up dates extracted into the REDCap database.

Data were imported into SAS V.9.4 (Cary, NC) for analysis. We first compared baseline characteristics of participants in each study arm, using chi-square or Fisher exact tests for categorical variables and ANOVA for continuous variables. To determine *feasibility*, we first calculated the proportion of participants in intervention arms who entered DSD. We then used data extracted from medical records to calculate intervals between clinical encounters by: (1) defining the DSD eligibility date as the first clinical appointment occurring ≥ 150 days after ART initiation; (2) defining the end date for the study window as the first encounter (clinical appointment or ART pick-up) occurring > 365 days after ART initiation; (3) calculating the mean number of days between each clinical appointment and each ART pick-up within the interval between ART initiation and DSD eligibility date; and (4) calculating the mean number of days between clinical appointments and ART pick-up within the interval between DSD eligibility date and the end date.

To determine *acceptability*, we separately calculated the average score for clinical and non-clinical satisfaction with care at 12-months, comparing scores by study arm (in an intention-to-treat [ITT] analysis) as well as in a *per protocol* analysis comparing those who advanced to DSD and those who did not (because of either change in clinical status or because they were not virally suppressed). We also tabulated instances when clinical staff overrode the study assignment.

To analyze *effectiveness*, we first conducted an ITT analysis, comparing the proportion of participants in each arm achieving 12-month viral suppression using a chi-square test. For this analysis, all participants were analyzed according to the randomization scheme, excluding three who were withdrawn within the first week after enrollment because they did not meet inclusion criteria. Participants who died or were lost to follow-up were considered to not be virally suppressed. Although the sample size of this pilot study was not sufficient to reach statistical power for non-inferiority, to assess this outcome, the estimated viral suppression rate for each arm was generated and compared using a logistic regression model adjusted for variables that were significantly different between arms at a level of *p* < 0.10. For each comparison in viral suppression rates, non-inferiority was confirmed if the lower bound of 95% confidence interval (CI) of the difference statistic was greater than the non-inferiority margin of 10%. To assess the impact of missing 12-month VL data on study outcomes, we repeated analyses above limited to participants who completed the 12-month visit (complete case analysis; *N* = 85). Finally, in a per protocol analysis, we compared viral suppression among participants completing all visits who advanced to DSD (*N* = 37), those who did not advance to DSD because they were not virally suppressed (*N* = 20), and those who were ineligible to advance to DSD because they were assigned to or crossed over to TAU (*N* = 28), using chi-square and logistic regression as above. Statistical significance for all tests was two-sided at *p* < 0.05.

## Results

### Participant characteristics

**Fig. 2:**
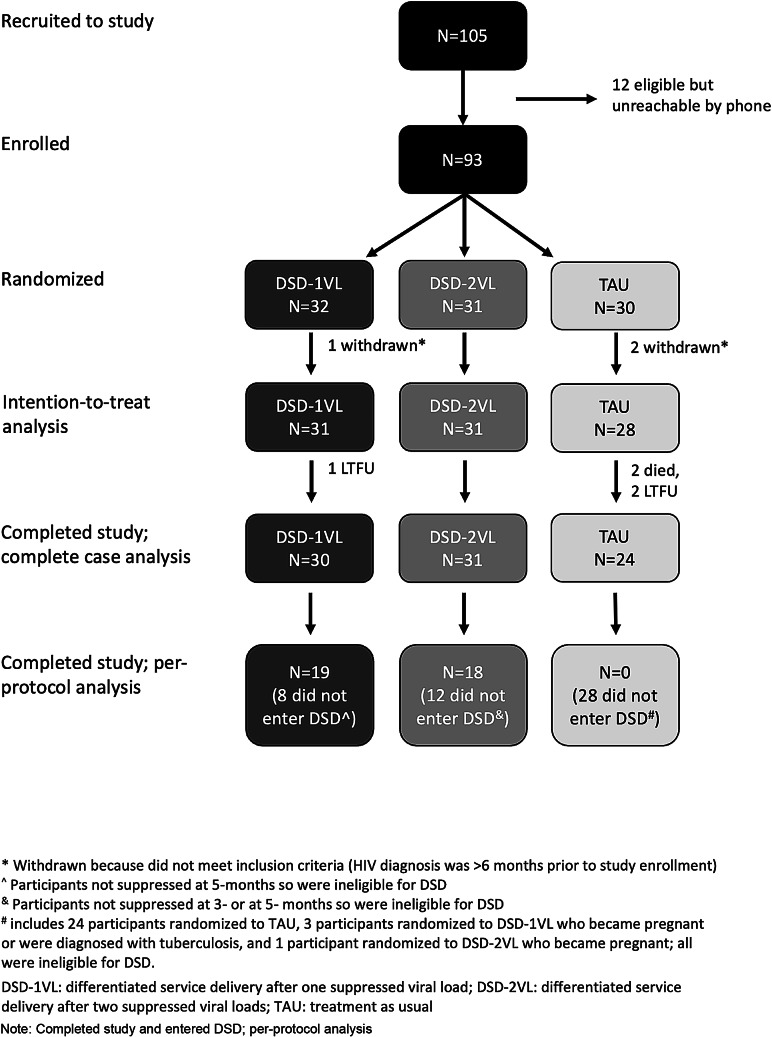
Flow chart showing study population, reasons for exclusion, and progress through study

During the recruitment period 105 patients were referred, of whom 12 (11%) were unreachable (Fig. 2). A total of 93 individuals were enrolled in the study; three were subsequently withdrawn and excluded from analyses after determining they did not meet inclusion criteria (HIV diagnosis was > 6 months prior to enrollment). Among the remaining 90 participants, 31 were randomized to DSD-1VL, 31 to DSD-2VL, and 28 to TAU. No minors under the age of 16 were enrolled. Arms were balanced with regards to participants’ baseline demographic characteristics, with the exception of education level and baseline internalized stigma score (Table 1). Over the course of the study, two participants died (both in TAU) and three were lost to follow-up (one in DSD-1VL, two in TAU); 85 completed the study.

**Table 1:** Baseline characteristics of participants (*N*=90)

	All participants n (%)	DSD-1VL (N=31) n (%)	DSD-2VL (N=31) n (%)	TAU (N=28) n (%)	*p*
Health center					0.98
Gikondo	36 (40.0)	13 (41.9)	13 (41.9)	10 (35.7)	
Kicukiro	17 (18.9)	6 (19.4)	5 (16.1)	6 (21.4)	
Remera	37 (41.1)	12 (38.7)	13 (41.9)	12 (42.9)	
Age, mean (SD)	30.8 (10.1)	29.97 (9.9)	33.35 (12.6)	28.89 (6.2)	0.20
Gender					0.81
Man	30 (33.3)	11 (35.5)	11 (35.5)	8 (28.6)	
Woman	60 (66.7)	20 (64.5)	20 (64.5)	20 (71.4)	
Marital status					0.89
Single	74 (82.2)	26 (83.9)	24 (77.4)	24 (85.7)	
Married	10 (11.1)	3 (9.7)	5 (16.1)	2 (7.1)	
Other (separated, divorced, widowed)	6 (6.7)	2 (6.5)	2 (6.5)	2 (7.1)	
Education					0.03
No schooling	21 (23.3)	3 (9.7)	10 (32.3)	8 (28.6)	
Completed primary	46 (51.1)	18 (58.1)	11 (35.5)	17 (60.7)	
Beyond primary (secondary, technical, or university)	23 (25.6)	10 (32.3)	10 (32.3)	3 (10.7)	
Median monthly household income in Rwandan Francs, in thousands (interquartile range)	30 (20-60)	40 (20-150)	30 (20-50)	35 (25-50)	0.49
Have health insurance	70 (77.8)	27 (87.1)	21 (67.7)	22 (78.6)	0.20
Employment status					0.43
Full time	14 (15.6)	6 (19.4)	6 (19.4)	2 (7.1)	
Part time or self-employed	49 (54.5)	16 (51.6)	14 (45.2)	19 (67.9)	
Not employed	27 (30.0)	9 (29.0)	11 (35.5)	7 (25.0)	
Number of people to whom HIV status was disclosed (SD)	1.5 (1.9)	1.8 (2.5)	1.5 (2.0)	1.1 (0.9)	0.38
Mean self-scored quality of life as measured by EQ-5D visual analog scale VAS score (SD)	42.9 (16.2)	45.0 (14.0)	45.5 (17.7)	37.9 (16.2)	0.14
Mean internalized stigma score, range 1-5 (SD)	1.9 (0.8)	2.0 (0.8)	1.6 (0.7)	2.1 (0.9)	0.06
Mean anticipated stigma score, range 1-5 (SD)	2.4 (0.7)	2.4 (0.7)	2.4 (0.7)	2.4 (0.8)	0.99
Mean enacted stigma score, range 1-4 (SD)	1.0 (0.11)	1.1 (0.2)	1.0 (0.1)	1.0 (0.0)	0.54
Mean days from care enrollment to ART initiation (SD)	2.2 (3.8)	2.4 (3.4)	2.5 (4.7)	1.8 (3.0)	0.72

SD: standard deviation

### Feasibility

Among 31 participants randomized to DSD-1VL, 22 (71%) were virally suppressed at 5 months (Table 2). Three participants who were suppressed were ineligible for DSD (two became pregnant, one was diagnosed with tuberculosis) and crossed over to TAU; the remaining 19 entered DSD. Among 31 participants in DSD-2VL, 19 (61%) were virally suppressed at both 3 and 5 months; one became pregnant and crossed over to TAU and the remaining 18 entered DSD. Among the 28 participants randomized into TAU, 19 (68%) were suppressed at 5 months; all continued in the guideline-based appointment and ART pick-up schedule.

**Table 2:** ART adherence and viral suppression, by study arm (*N*=90)

	All participans (N=90)	DSD-1VL (N=31)	DSD-2VL (N=31)	TAU (N=28)	*p*
	*Days (SD)*	*Days (SD)*	*Days (SD)*	*Days (SD)*	
*ART adherence*					
Self-reported number of days of ART missed in prior 30 days, at 6-month visit	1.1 (3.5)	0.9 (2.0)	1.6 (5.5)	0.6 (1.1)	0.55
Self-reported number of days of ART missed in prior 30 days, at 12-month visit	0.9 (3.4)	0.8 (1.2)	1.1 (5.4)	0.6 (1.1)	0.85
	*n (%)*	*n (%)*	*n (%)*	*n (%)*	
*Viral suppression*					
Virally suppressed, 3-month viral load^^^	23 (74.0)	-	23 (74.0)	-	-
Virally suppressed, 5-month viral load	62 (68.9)	22 (71.0)	21 (67.7)	19 (67.9)	0.80
Eligible to enter DSD schedule^&^	37 (59.7)	19 (61.3)	18 (58.1)	-	0.796
Virally suppressed, 12-month viral load	69 (76.7)	25 (80.7)	25 (80.7)	19 (67.9)	0.41
Virally suppressed, 12-month viral load, among those completing study^*^	69 (82.1)	25 (83.3)	25 (80.7)	19 (82.6)	1.00

ART: antiretroviral therapy; DSD: differentiated service delivery

^only includes participants in DSD-2VL

& only includes participants in DSD-1VL and DSD-2VL; does not include 3 participants in DSD-1VL and 1 participant in DSD-2VL who were ineligible for DSD appointment schedule because of pregnancy or tuberculosis; p-value for chi-square test comparing DSD-1VL and DSD-2VL

*denominators are *N*=30 (DSD-1VL), *N*=31 (DSD-2VL), *N*=24 (TAU)

Overall, the mean interval between clinical appointments was 90 days (standard deviation [SD] 38) between ART initiation and the DSD eligibility date, and 132 days (SD 47) between the DSD eligibility date and the end of the study (*p* < 0.001) (Table 3). In the per-protocol analysis, among 37 participants who advanced to DSD, mean clinical appointment interval between the DSD eligibility date and the end of the study was 154 days (SD 52), compared to 104 days (SD 29) among participants in intervention arms who were not virally suppressed and did not advance to the DSD schedule and 126 days (SD 39) among those not eligible to advance (*p* < 0.001). The overall mean interval between ART pick-ups was 34 days (SD 10) between ART initiation and the DSD eligibility date, and 61 days (SD 40) between the DSD eligibility date and the end of the study (*p* < 0.001). Among 37 participants in DSD-1VL and DSD-2VL who advanced to the DSD appointment schedule, mean ART pick-up interval between the DSD eligibility date and the end of the study was 88 days (SD 45), compared to 42 days (SD 22) among participants who were not virally suppressed and did not advance to the DSD schedule and 40 days (SD 19) among those not eligible to advance (*p* < 0.001).

**Table 3:** Intervals between clinical appointments and ART pick-ups, by whether participants advanced, did not advance, or were not eligible to advance to DSD (*N*=90)

	All participants Mean (SD)	Advanced to DSD^ Mean (SD)	Did not advance to DSD^&^ Mean (SD)	Not eligible to advance to DSD* Mean (SD)	*p*
Interval between clinical appointments from ART initiation to DSD eligibility date, days	90.0 (38.4)	85.7 (30.3)	81.4 (21.9)	102.1 (53.4)	0.136
Interval between clinical appointments from DSD Eligibility Date to Last Date, days	132.2 (47.0)	154.0 (51.6)	103.6 (28.7)	126.2 (39.1)	<0.001
Interval between pharmacy pick-ups from ART initiation date to DSD Eligibility Date, days	34.2 (9.5)	32.8 (5.6)	36.9 (15.5)	34.2 (7.8)	0.322
Interval between pharmacy pick-ups from DSD Eligibility Date to Last Date, days	60.6 (40.0)	87.8 (44.6)	41.8 (22.4)	40.0 (18.6)	<0.001

### Acceptability

In the overall sample, 12-month participant-level acceptability was high for both clinical (mean score 3.8 out of 5, SD 0.3) and non-clinical (4.1, SD 0.4) elements of care and did not differ significantly across study arms (p 0.91 and 0.78, respectively). Similar results were observed comparing participants who advanced to DSD (mean scores of 3.8 and 4.1, for clinical and non-clinical elements respectively) and those who did not (mean scores of 4.1 and 3.8). Health center staff partially overrode the study assignment for 13 of 37 participants (35%) who were eligible to advance to DSD. Twelve were effectively classified in the “Stable B” category outlined in national guidelines (clinical appointments every 3 months, quarterly ART pick-ups) rather than “Stable A” (clinical appointments every 6 months, quarterly ART pick-ups); one participant continued to attend quarterly clinical appointments and monthly ART pick-ups. No study-related adverse events occurred during the study period.

### Effectiveness

In the ITT analysis, 25 of 31 participants (81%) randomized to DSD-1VL, 25 of 31 participants randomized to DSD-2VL (81%), and 19 of 28 participants randomized to TAU (68%) were virally suppressed at 12 months (*p* = 0.41). In a multivariable analysis accounting for differences in baseline educational attainment and internalized stigma, odds of viral suppression were higher in DSD-1VL (adjusted odds ratio [aOR] 1.12, 95% confidence interval [CI] 0.90–1.41) and DSD-2VL (aOR 1.11, 95% CI 0.88–1.38) compared to TAU but were not statistically significant. The differences between the estimated arm-specific viral suppression rates (generated from results of the logistical regression models) did not meet significance for non-inferiority. In the complete case analysis, 12-month viral suppression was achieved among 83% of participants in DSD-1VL, 81% in DSD-2VL, and 83% in TAU (*p* = 1.00). Finally, in the per protocol analysis, among 37 participants who entered DSD (in either DSD-1VL or DSD-2VL), 36 (97%) were suppressed; of the 48 not in DSD, 33 (69%) were suppressed (p = < 0.001).

## Discussion

In one of the first studies to empirically compare early (6-months after ART initiation) entry into a facility-based DSD model after one or two suppressed VLs to the standard of care (DSD after 12 months on ART and two suppressed VLs), we found that these strategies were largely feasible, acceptable to patients and similar with respect to 12-month viral suppression. Findings from this pilot RCT support the most recent WHO guidelines recommending implementation of less-intensive DSD models for PWH established on ART, defined as receiving ART for at least six months with at least one suppressed VL [9].

Our results suggest that early DSD is feasible, although not for all PWH. Among participants who entered DSD, the average interval between clinical appointments and ART pick-ups during study months 6–12 were much greater than among participants not in DSD and were similar to appointment schedules specified in the study protocol. These findings suggest that advancing eligible PWH to spaced-out appointments at 6-months after ART initiation is achievable. Among participants randomized to DSD-1VL and DSD-2VL, 71% and 58% were eligible to enter DSD based on one or two suppressed VLs, respectively. These proportions are substantially lower than the overall rate of viral suppression among PWH on ART in Rwanda of 90.1% [19]. Nonetheless, these figures are comparable to benchmarks used in recent studies examining HIV health services interventions including point-of-care VL testing and HIV self-testing [20, 21]. All participants in this study were on a dolutegravir-based regimen which may be expected to result in viral suppression for most PWH by 3 months after initiation [22, 23]. Studies in Rwanda and elsewhere in SSA demonstrate that stability on ART, including achieving viral suppression, can be challenging early in the course of HIV secondary to stigma and adjusting to the diagnosis and lifelong medications [13, 24, 25]. Our findings suggest that early entry into DSD may not be possible for all PWH and that some may require a longer period of more intensive care at the beginning of their treatment course.

We observed no significant differences in acceptability with care between study arms or when comparing participants who entered DSD and those who did not, suggesting that early DSD is acceptable to Rwandan PWH. Our results are similar to research showing similar quality of life among people enrolled in DSD and those in traditional models [26], although some studies have described lower satisfaction with care among patients in DSD models for shorter compared to longer periods of time [27]. We found that acceptability did not differ between study arms or between those enrolled in DSD and those in TAU, suggesting that differences were not related to frequency of encounters.

Clinicians at study health facilities partially overrode the study protocol for 13 of the 37 patients who were eligible for DSD, as reflected in the average inter-clinical appointment interval substantially below the expected 180 days. On review of medical records of these 13 patients, 12 were classified as “Stable B,” a designation that allows quarterly ART pick-ups while also continuing to require clinical appointments every 3 months. This deviation from the protocol may reflect hesitancy on the part of clinical staff to fully implement DSD early in patients’ treatment course, despite enthusiasm from health facility leadership and training of staff prior to beginning enrollment. Prior studies have shown lack of buy-in by providers as a barrier to widespread DSD implementation [28, 29], and suggests that interventions aimed at healthcare staff may be needed as early DSD programs are scaled up.

In the ITT analysis, a slightly higher proportion of participants randomized to DSD-1VL and DSD-2VL were virally suppressed at 12 months, although this finding did not reach statistical significance. Studies of DSD programs among PWH more established on ART (i.e. >12 months) have largely reported that viral suppression was no worse or even better among patients in facility-based DSD programs compared to those in routine care [5]. Though fewer data on early DSD outcomes exist, a study of South African patients advanced to DSD after being on ART between 6 and 12 months (average duration before switch: 10 months) found similar rates of viral suppression to patients in routine care [30]. While our pilot study was underpowered to detect non-inferiority for 12-month viral suppression, our findings suggest that even six months on ART is sufficient to reach stability necessary to space out clinical encounters, supporting recent WHO guidelines. Importantly, we observed no difference in 12-month viral suppression among those in DSD-1VL and those DSD-2VL, suggesting that one VL measurement may be sufficient to determine stability on ART. These findings can be particularly useful in considering health system costs, as one concern about early DSD would be the added “start up” cost in the first year if multiple VL measurements were required.

Several important strengths as well as limitations of this pilot study are worth noting. This was among the first investigations to empirically compare early entry into DSD to the standard of care. Notably, our design included two intervention arms, allowing us to also compare differences in 12-month viral suppression among those who entered DSD after one versus two suppressed viral loads. Enrolling participants in the study at the time of ART initiation, rather than limiting inclusion to patients suppressed at 5 months, allowed us to examine the overall feasibility of early DSD, compare feasibility and effectiveness of DSD-1VL and DSD-2VL, and resulted in a sample more representative of newly-diagnosed PWH in Rwanda. The main limitation of this study design was a reduction in statistical power to detect differences in effectiveness across study arms, as a substantial proportion of participants assigned to intervention arms did not enter DSD. We encountered difficulty in measuring appointment adherence (a prespecified outcome) because of inconsistent documentation of scheduled appointments, and therefore were not able to report on this outcome. The study was unblinded, and participants entered DSD at the discretion of treating clinicians, which may have introduced bias; specifically, the classification of 12 participants to a “Stable B” schedule with more frequent clinic appointments may have contributed to the very high 12-month viral suppression among those who entered DSD. Given its pilot nature, the study was not sufficiently powered to determine non-inferiority within the margin of significance. The study period coincided with peak months of the COVID-19 pandemic, which disrupted HIV care in Rwanda and may have impacted clinical decision-making by nurses. Finally, Rwanda’s highly functional HIV care service delivery system and lower HIV prevalence than in much of SSA may limit the generalizability of our findings.

Supported by the newest WHO guidelines, multiple settings have implemented facility-based DSD models at 6 months after ART initiation [10, 31, 32]. Changes related to the COVID-19 pandemic have also led to more widespread implementation of DSD even for newly-diagnosed PWH [8, 33–35]. Our results suggest that DSD at 6 months after ART initiation is acceptable to and feasible for many newly-diagnosed PWH, although some patients may require additional time to reach viral suppression. Examining the impact of early DSD on viral suppression in a larger, adequately powered study is necessary to fully understand the effectiveness of this strategy. Nonetheless, our findings support calls for tailored DSD implementation as part of a toolbox of patient-centered approaches to end the HIV epidemic [13, 36].

### Electronic supplementary material

Below is the link to the electronic supplementary material.

Supplementary Material 1

## Data Availability

The data that support the findings of this study may be made available for researchers who meet the criteria for access to confidential data upon reasonable request from the corresponding author, JR. The data are not publicly available because permission for public dissemination of raw data was not solicited from the Rwanda National Ethics Committee at the time of initial review of the protocol.
